# Diagnosis and treatment of infectious vaginitis: Proposal for a new algorithm

**DOI:** 10.3389/fmed.2023.1040072

**Published:** 2023-02-09

**Authors:** José Eleutério, Adriana Bittencourt Campaner, Newton Sergio de Carvalho

**Affiliations:** ^1^Department of Health for Women, Children, and Adolescents, Faculty of Medicine, Federal University of Ceará, Fortaleza, Brazil; ^2^Department of Gynecology and Obstetrics, Faculty of Medical Sciences of Santa Casa de São Paulo, São Paulo, Brazil; ^3^Department of Gynecology and Obstetrics, Infectious Diseases in Gynecology and Obstetrics Sector, Federal University of Paraná, Curitiba, Paraná, Brazil

**Keywords:** Vaginitis, bacterial vaginosis, Genital infection, Candidiasis, trichomoniasis

## Abstract

**Background:**

Vaginitis is the most common gynecologic diagnosis in primary care, and most women have at least one episode during their lives. The need for standardized strategies to diagnose and treat vaginitis, both in primary care and among gynecologists, is emphasized. The Brazilian Group for Vaginal Infections (GBIV, acronym in Portuguese) aimed to update the practical approach to affected women by reviewing and discussing recent literature, and developing algorithms for diagnosis and treatment of vaginitis.

**Methods:**

A literature search within biomedical databases PubMed and SCieLo was conducted in January 2022. The available literature was evaluated by three experienced researchers, members of the GBIV, to summarize the main data and develop practical algorithms.

**Results and conclusion:**

Detailed algorithms were developed with the main goal to improve gynecological practice considering different scenarios and access to diagnostic tools, from the simplest to the most complex tests. Different age groups and specific contexts were also considered. The combination of anamnesis, gynecological examination, and complementary tests remains the basis of a proper diagnostic and therapeutic approach. Periodic updates of these algorithms are warranted as new evidence becomes available.

## Introduction

Vaginal symptoms, such as discharge, odor, and itching are frequent, especially in women of reproductive age (1). Vaginitis is the most common gynecologic diagnosis in primary care, and most women have at least one episode during their lives (2). It has been demonstrated that vaginitis may have a negative impact on quality of life, including embarrassment, and anxiety, particularly in women with recurrent episodes (2).

Bacterial vaginosis (BV), the most common cause of vaginal discharge, has also been associated with several potential complications, including a higher risk of preterm delivery, and an increased risk of acquiring human immunodeficiency virus (HIV) infection, human papillomavirus (HPV) infection, other sexually transmitted diseases, and pelvic inflammatory disease (3). BV is the cause in 40–50% of cases with an identified etiology (2). Vulvovaginal candidiasis (VVC) and trichomoniasis account for 20–25% and 15–20% of cases in premenopausal women, respectively (4). Non-infectious causes are less common and account for the remaining cases (2, 4). Mixed vaginitis, along with VVC and BV, is considered an important differential diagnosis; according to Lowe et al. (5) in a case series considering 545 women with signs and symptoms of vulvovaginitis, BV, VVC, and mixed vaginitis accounted for 42, 14, and 16% of all patients, respectively.

In the context of the high prevalence and importance of BV and vaginitis, the need for standardized strategies to diagnose and treat these conditions, both in primary care and among gynecologists, is emphasized. The Brazilian Group for Vaginal Infections (GBIV, for its acronym in Portuguese) aimed to help physicians update the practical approach to affected women by reviewing and discussing recent literature, and developing algorithms for diagnosis and treatment in different healthcare levels of assistance.

## Methods

To identify the approaches employed in diagnosing and treating BV and vaginitis to develop the proposed algorithms, a literature search within biomedical databases PubMed and SCieLo was conducted in January 2022. The search terms were “bacterial vaginosis” and/or “vaginitis” AND “clinical reasoning,” “differential diagnosis,” “diagnostic,” “treatment,” “approach.” Portuguese and Spanish equivalent words were also used. Wildcards were allowed.

Papers published until the cutoff date that contained the query in the title or the abstract were selected. Only articles in English, Spanish, or Portuguese indexed as original articles or reviews were considered. After reading the abstracts, the articles not related to clinical and/or laboratory diagnosis or pharmacological or non-pharmacological treatments were excluded. Subsequently, references of the publications were searched for additional relevant articles.

The available literature was evaluated by three experienced researchers, members of the GBIV, to summarize the main data and develop practical algorithms.

## Results

### Diagnostic tools

Normal physiological vaginal discharge changes with the menstrual cycle and may increase premenstrually, at the time of ovulation and in women who start hormone replacement treatment or hormonal contraception (6). In contrast, vaginal infection and microbiota alterations can be associated with different discharge forms, local irritation, pruritus (itching), and pain (6). Anamnesis should include aspects related to vaginal discharge (color, consistency, unpleasant odor, and modifications), sexual behavior and practices, type of contraceptive method, vaginal hygiene practices, date of last menstrual period, use of topical or systemic medication, and other potential irritating agents (6). The presence of comorbidities like diabetes mellitus and HIV infection should also be evaluated (6).

During the gynecological examination, the healthcare professional must identify the characteristics of vaginal fluid during the speculum examination. The presence of alterations, including colpitis, ulcers, fissures, edema, and erythema should be highlighted (6).

Office-based tests (vaginal pH, amine test, saline, and 10% KOH microscopy) are easily available and may frequently help to diagnose common forms of vaginitis; however, office testing may have poor performance characteristics (7).

Even though anamnesis, gynecological examination, and office-based tests remain the primary diagnostic tools, current evidence suggests that clinical diagnoses may be poorly correlated with laboratory findings (8). Therefore, laboratory tests are warranted to determine the etiology of vaginal symptoms (9). Frequent pathogens are often identified after examination of the vaginal contents by bacterioscopy and the Pap smear (although the test is not primarily for this type of diagnosis). For *Candida* and *Trichomonas*, the sensitivity rate of the Pap test is 50% (7). More recently, liquid-based cytology and DNA probe tests were also added to the diagnostic tools for vaginal symptoms (7). Molecular tests directed to BV diagnosis, *Candida*, and *T. vaginalis* can improve diagnostic accuracy and reduce the time to diagnosis when compared to conventional culture techniques (9, 10).

### Specific vaginitis causes

Bacterial vaginosis is a disorder associated with a decrease in peroxide-producing lactobacilli and an increase in the number of anaerobic and facultative bacteria (11), including short Gram-variable bacilli, short Gram-negative bacilli, and anaerobic Gram-negative cocci, with variations, mainly *Gardnerella, Atopobium, Prevotella, Megasphaera, Leptotrichia, Sneathia, Bifidobacterium, Dialister, Mobiluncus, Ureaplasma*, and *Mycoplasma* (6). Even though BV is the most frequent cause of vaginal discharge in woman of reproductive age (2), it is usually underdiagnosed in the general practitioner setting (8). Although some cases are asymptomatic, BV tends to present with a thin discharge, characterized by a foul smell in alkaline environments (including semen, blood, and 10% KOH) (9). BV is diagnosed using either Amsel’s criteria or Nugent score; due to methodological differences between these two diagnostic techniques, accuracy results may vary when these methods are compared (12).

Vulvovaginal candidiasis is characterized by erythema, vulvar fissures, clumpy discharge, vulvar edema, lesions related to intense scratching, and eventually dyspareunia and dysuria due to irritation and local lesions. Diagnosis must be confirmed through laboratory examinations, mainly microscopic examination of fresh vaginal content and Gram-stained vaginal smear bacterioscopy. The culture is essential in cases of recurrent VVC to identify *Candida* species (6). Recently, molecular biology tools [mainly polymerase chain reaction (PCR) based on the multiplex platform] were developed; these tools are characterized by high sensitivity rates and faster results for identifying *Candida* species, and may be indicated instead of culture (13).

Vulvovaginal candidiasis is defined as non-complicated when all the following criteria are met: mild/moderate symptoms and rare frequency, *C. albicans* as an etiologic agent, and lack of comorbidities. In contrast, complicated VVC is diagnosed whether at least one of these criteria is met: intense symptoms; recurrence (≥3 episodes/year); pregnancy; *C.* non-*albicans* as etiologic agent; and comorbidities such as diabetes mellitus or HIV (6, 14).

Most cases of *Trichomonas* infections are asymptomatic, precluding diagnosis or treatment. Symptomatic women may present with greenish-yellow and foamy intense vaginal discharge, with a fetid odor. Pruritus, bleeding during intercourse, dyspareunia, vulvar edema, and even urinary symptoms may be associated. Microscopic examination of fresh vaginal content in saline may reveal moving parasites, and Papanicolaou or Giemsa techniques may also identify this agent (6). Nevertheless, the gold standard method for identification of *T. vaginalis* is real-time PCR (14).

In addition to the previously described clinical syndrome, mixed vaginitis is characterized by two concurrent pathogens causing the disease. Symptoms may vary depending on the microorganisms involved; nevertheless, the etiological diagnosis is essential, mainly using multiplex platforms. Study of microorganisms by examination of fresh or stained (Gram) vaginal fluid and Pap smear could also be used. The most frequent mixed vaginitis is concurrent BV and VVC (15). According to Qui et al. (16) there is significant variability in the prevalence of mixed vaginitis, which includes BV/VVC, BV/aerobic vaginitis (AV), and VVC/AV, among others. Studies on this condition are still preliminary, and many cases of mixed vaginitis are underdiagnosed.

Other causes of vaginitis are less common; however, it is essential for healthcare providers to diagnose and treat these conditions properly. Differential diagnoses include cytolytic vaginosis, desquamative inflammatory vaginitis (DIV), and AV. Cytolytic vaginosis is the consequence of an aerobic imbalance of vaginal microbiota, with an increase in the lactobacilli population leading to cytolysis. Patients usually report pruritus and discharge similar to VVC, and laboratorial diagnosis is based on Gram bacterioscopy (17).

Desquamative inflammatory vaginitis is an ill-defined condition causing purulent discharge, vestibule-vaginal irritation, and dyspareunia. DIV is characterized by an increase in inflammatory cells and parabasal epithelial cells (immature squamous cells). Vaginal microbiota is abnormal and pH is elevated (>4.5) (18). DIV is defined by some authors as a more severe picture of aerobic vaginitis, a condition associated with aerobic microorganisms, mainly group B streptococci and *E. coli*, even though these microorganism are not causal of AV/DIV. Its characteristics are different from those of BV and elicit an important host response. AV diagnostic criteria, as described by Donders et al. (19) were based on phase contrast microscopy of vaginal fluid by developing a score including the lactobacilli grade, the leukocytes count, the presence of toxic leukocytes, the microbiota morphotypes and the proportion of parabasal cells. A composite score of 1–2 represents normality. Respective scores of 3–4, 5–6, and 6–10 represent slight, moderate or severe AV. According to the authors, the severe form of AV would be identical to DIV (19).

Finally, atrophic vaginitis is associated with low estrogen levels and may cause symptoms like vaginal discharge, pruritus, and dyspareunia. The diagnosis is based on Pap smear and Gram bacterioscopy (1).

It is also extremely important to exclude cervicitis, as this clinical condition may also cause symptoms and lead to local changes that may favor the development of vaginitis. The main agents causing cervicitis include *Chlamydia trachomatis, Neisseria gonorrhoeae*, and *Mycoplasma genitalium* (7).

### Diagnostic algorithm

**FIGURE 1:**
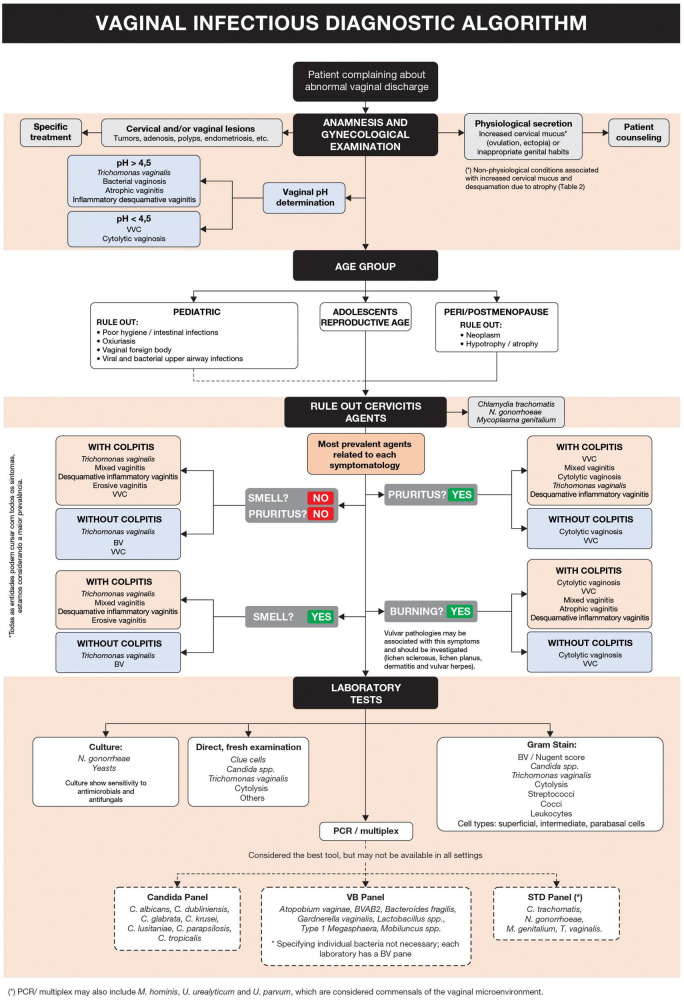
Vaginal infectious diagnostic algorithm.

The proposed diagnostic algorithm is shown in Figure 1. Complementary information for specific and differential diagnosis is summarized in Tables 1, 2, respectively (6–13).

**TABLE 1:** Laboratory tests for diagnosing infectious microorganisms that may be observed in vaginitis and cervicitis (*Chlamydia trachomatis*, *Neisseria gonorrhoeae*, and *Mycoplasma* spp).

Agent	Test	Results	Sensitivity
*Candida* spp	Vaginal pH	<4.5	–
	Fresh sample microscopy	Morphotype observation	60%
	Gram stain	Morphotype observation	80%
	Amine test	Negative	–
	Culture (Sabouraud and Chromoagar): allows antifungal sensitivity determination	Specific species identification	>95%
	PCR/Multiplex–*Candida albicans* and non-*albicans* panel	Specific species identification	>95%
*Trichomonas vaginalis*	Vaginal pH	>4.5	–
	Wet mount microscopy	Parasite observation	50% without colpitis* 80% with colpitis
	Gram stain	Morphotype observation	<40%*
	Amine test	Usually positive	–
	PCR/Multiplex	Specific species identification	>95%
*Chlamydia trachomatis*	PCR/Multiplex	Specific species identification	>95%**
*N. gonorrheae*	Gram stain	Intraleukocytic Gram-negative diplococci	∼70%***
	Culture (Thayer–Martin)	Colony growth	∼90%
	PCR/Multiplex	Specific species identification	>95%
*Mycoplasma* spp.	PCR/Multiplex	Specific species identification	>95%
Mixed vaginitis	Wet mount microscopy	Clue cell identification, associated with *Candida* morphotypes	70%
	Gram stain	*Candida* morphotype identification (score 7–10)	90%
	PCR/Multiplex (BV + *Candida*)	Specific species identification	>95%

*Cases without colpitis are usually associated with the pseudocyst form of *T. vaginalis* (parasites are immobile and smaller than the active form). **Persistent discharge may be associated with interference in the microbiota caused by *C. trachomatis* cervicitis. ***If the sample is taken from the cervix, the sensitivity is lower than 50%.

**TABLE 2:** Available tests for the differential diagnosis of less frequent causes of vaginitis.

Disorder	Test	Result	Sensitivity
Cytolytic vaginosis	Vaginal pH	<4.5	–
	Wet mount microscopy	Cytolysis without inflammation	70%
	Gram stain	Cytolysis without inflammation	90%
	Pap smear*	Cytolysis without inflammation	–
	Fungi culture	Negative	–
	PCR/Multiplex for pathogens (Table 1)	Negative	–
Aerobic vaginitis/desquamative, inflammatory vaginitis	Vaginal pH	>4.5	–
	Fresh sample microscopy	Identification of parabasal cells and inflammatory infiltrates	50%
	Gram stain	Identification of parabasal cells and inflammatory infiltrates	50%
Atrophic vaginitis	Vaginal pH	>4.5	–
	Gram stain	Identification of parabasal cells and sparse microbiota composed predominantly of cocci	–
	Pap smear	Identification of parabasal cells and “blue blobs”	>90%

*Used for cervical cancer screening, but it can show pathogens.

### Treatment

**FIGURE 2:**
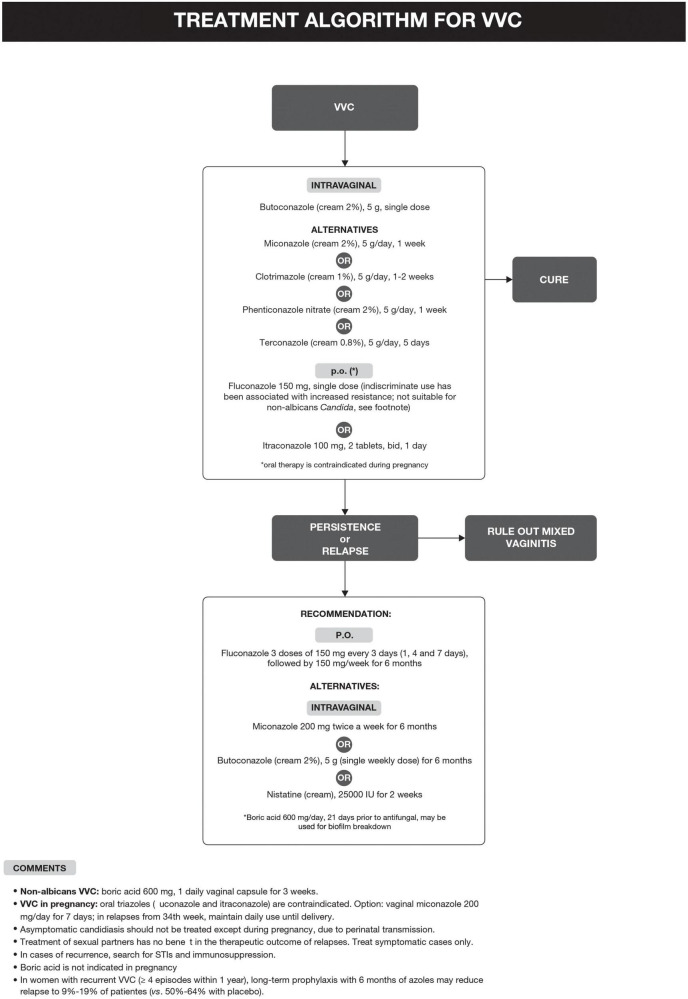
Treatment algorithm for vulvovaginal candidiasis (VVC).

The treatment algorithm for VVC is shown in Figure 2.

**FIGURE 3:**
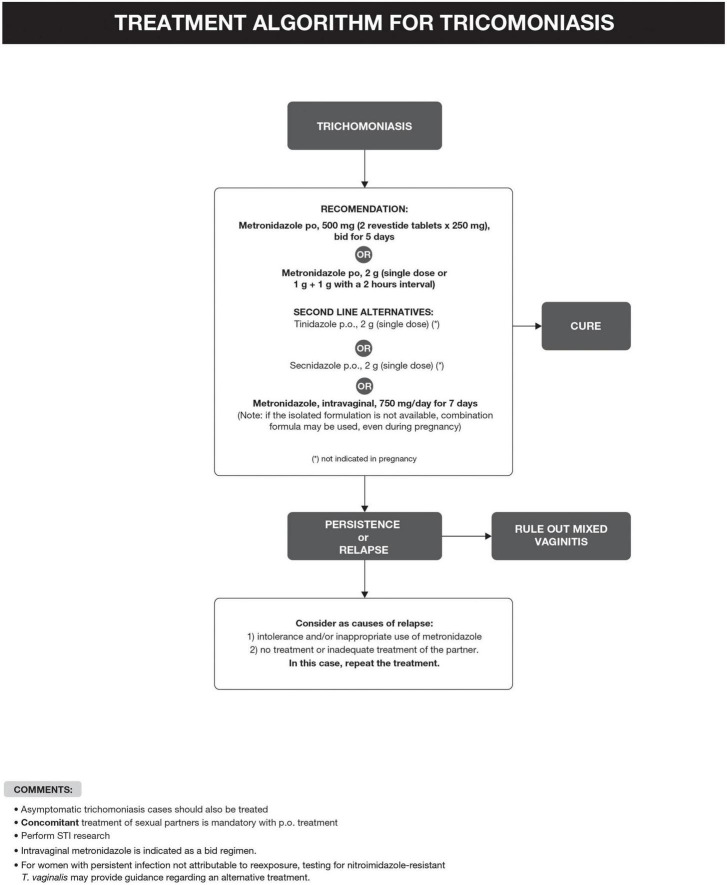
Treatment algorithm for trichomoniasis.

The recommended treatment of *T. vaginalis* is summarized in Figure 3 (6, 14–31). Taking into account a recurrence rate of 5–31%, it is important to assess partners’ treatment, exposure to new partners, and the possibility of therapeutic failure, which seems more frequent in women treated with a single dose regimen and with HIV infection (6, 16).

**FIGURE 4:**
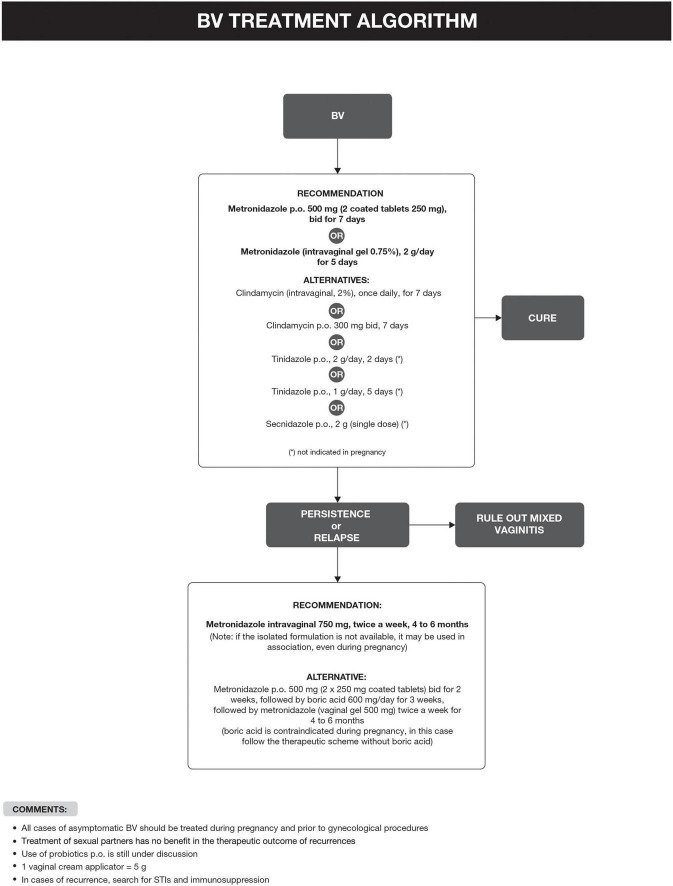
Bacterial vaginosis (BV) treatment algorithm.

**FIGURE 5:**
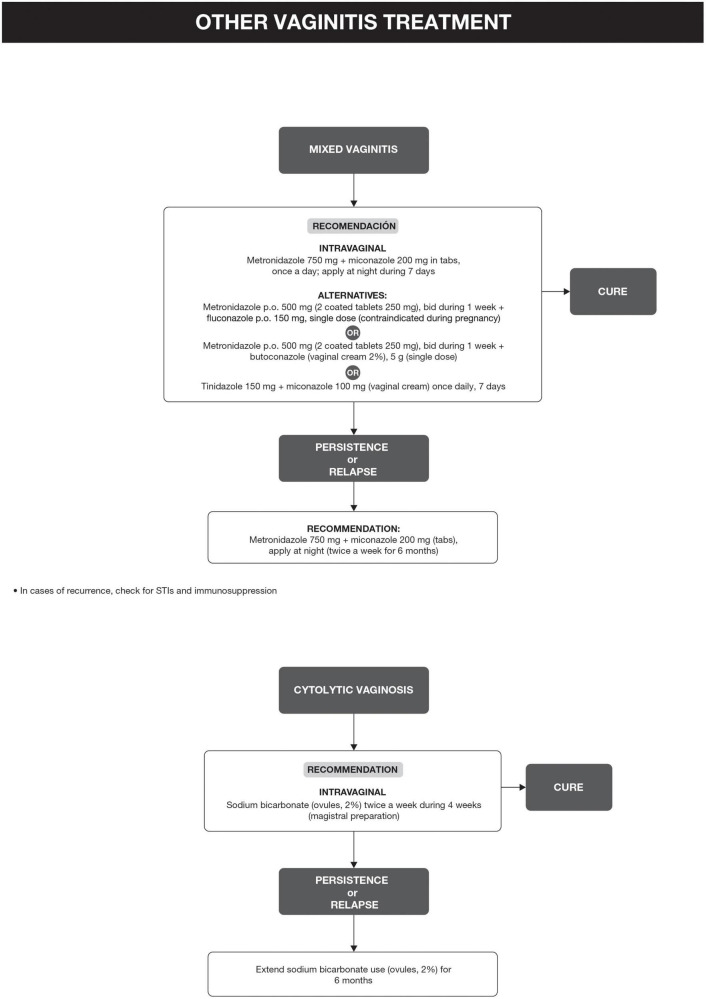
Other vaginitis treatment.

**FIGURE 6:**
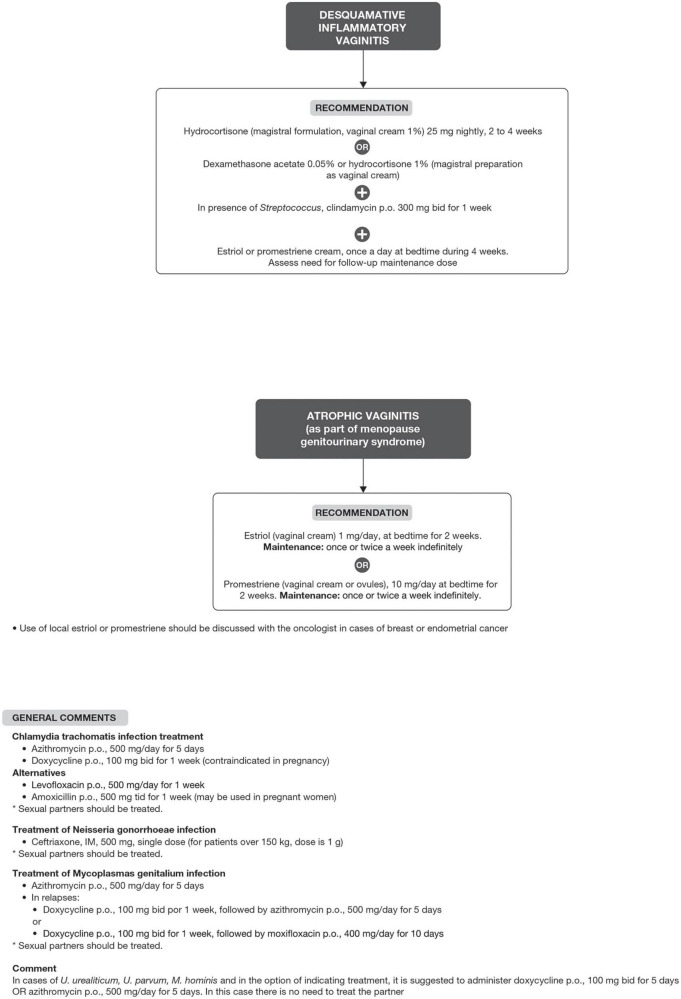
Treatment for aerobic vaginitis/desquamative inflammatory vaginitis, atrophic vaginitis and cervicitis.

Bacterial vaginosis treatment is recommended for all symptomatic and asymptomatic women with the potential risk of complications, such as previous gynecological surgeries or procedures. Recurrence of BV after treatment is common. Some factors have been associated with the lack of therapeutic response, including frequent unprotected sexual intercourse, vaginal douches, altered immune response, the occurrence of biofilms, and bacterial resistance to imidazoles and clindamycin (6, 29). The proposed treatment algorithm is shown in Figure 4 (6, 15, 18, 20–26). Treatment for other causes of vaginal symptoms (6, 27, 28) is summarized in Figure 5. Treatment for aerobic vaginitis/desquamative inflammatory vaginitis, atrophic vaginitis and cervicitis is summarized in Figure 6 (6, 14–31).

## Discussion

The most frequent causes of vaginal discharge are BV, VVC, mixed vaginitis, and trichomoniasis. Nevertheless, clinical symptoms may be poorly correlated with etiology (9). Many women presenting with vaginitis are assisted by general practitioners, and both overdiagnosis of VVC and underdiagnosis of BV are reported in that setting (8).

Physicians need to make sequential decisions in their clinical practice. Algorithms are a useful tool, as their graphic format sets a stepwise procedure for making decisions about both diagnosis and treatment of a clinical problem (32). Algorithms are especially helpful because of the astonishing amount of medical evidence, preferences, and healthcare scenarios to consider before arriving at a final diagnosis and treatment (33). Decision-making aids, like algorithms, are balanced sources of information outlining treatment options for a particular health condition (32).

In this narrative review, several guidelines and observational trials have been analyzed, including important international (3–5, 7, 34–36) and regional recommendations (6, 9, 11, 13). However, the optimal care for any particular patient requires not only understanding the relevant published evidence, but also integrating experiential medical knowledge, and elucidating patient’s goals and values, all within the intrinsic complexity of local healthcare systems (37).

Members from the GBIV have analyzed the main available evidence in the context of their broad clinical experience; as a result, detailed algorithms have been developed with the main goal to improve gynecological practice considering different scenarios and access to diagnostic tools, from the simplest to the most complex tests. Different age groups and specific contexts (pregnancy, drug availability) have also been considered. The combination of anamnesis, gynecological examination, and complementary tests remains the basis of a proper diagnostic and therapeutic approach. Periodic updates of these algorithms are warranted as new evidence becomes available.

## Author contributions

All authors contributed to the study conception, design, data collection, analysis, material preparation, and read and approved the final manuscript.
